# Magnetism of the Blood

**Published:** 1886-10

**Authors:** 


MAGNETISM OF THE BLOOD.—Magini and Maggiorani have reported to the Royal Academy of Medicine of Rome the results of their experimental study of magnetism of the blood. Their conclusions are as follows:

1. That the blood is normally diamagnetic.

2. That there are substances capable of diminishing the physiological diamagnetism of the blood, and may even render it paramagnetic.

3. That there is a relation between the rapidity or slowness of coagulation of the blood and its degree of diamagnetism.

4. That some mineral waters are paramagnetic and others diamagnetic, probably depending upon the predominant saline element. These mineral waters, when taken internally, modify the magnetic properties of the blood.

5. That carbonic acid constantly diminishes the diamagnetism of the blood in pigeons and probably in other animals.

6. That the alkaloids modify the diamagnetic power of normal blood.

7 That temperature has a great influence upon the magnetic properties of bodies, being capable of rendering diamagnetic bodies paramagnetic, and conversely.—*Bull. della Real. Accad. Med.*

